# Diversity, Distribution, and Phenotypic Characterization of Cultivable Wild Yeasts Isolated from Natural Forest

**DOI:** 10.12688/f1000research.160250.1

**Published:** 2025-01-17

**Authors:** Teshome Tadesse, Degife Dese, Anbessa Dabassa, Ketema Bacha

**Affiliations:** 1 Biology, Mettu University, Metu, Oromia, 318, Ethiopia; 2 Biology, Jimma University College of Natural Sciences, Jimma, Oromia, 378, Ethiopia

**Keywords:** Distribution, Ethiopia, Forest, Stress Tolerant Yeast, Wild Yeast

## Abstract

**Background:**

Yeasts are unicellular fungi that inhabit a variety of environments including plant surfaces, water, soil, and animal hosts. However, limited research has been conducted on soil and plant associated yeasts in Africa, with most studies originating from developed regions.

**Methods:**

This study explored the diversity, distribution, and phenotypic characterization of cultivable wild yeast in samples from rhizosphere soil, leaves, litter, and tree bark collected from South West Ethiopia. Yeast isolates were characterized using morphological, physiological and biochemical methods, Stress-tolerant yeast species were identified using Matrix-Assisted Laser Desorption Ionization-Time of Flight (MALDI-TOF).

**Results:**

Based on morphological, physiological, and biochemical analyses, a total of 15 yeast genera were identified from 23 plant species. Predominant yeast species included *Candida* spp., *Saccharomyces* spp., *Meyerozyma* spp., *Pichia* spp., *Geotrichum* spp., and *Hanseniaspora* spp. Plant species with the highest yeast diversity were *Ficus vasta*, *Ficus exasperata*, *Ficus sycomorus*, *Cordia africana*, and *Ritchiea albersii.* Bark samples yielded more yeast isolates than rhizosphere soil, litter, and leaves. Stress-tolerant species such as *Saccharomyces cerevisiae*, *Candida pelliculosa*, *Meyerozyma guilliermondii*, *Pichia kluyveri*, and *Trichosporon asahii* were identified using MALDI-TOF. Correlation analysis revealed no significant relationship between yeast populations in bark and leaf samples or between rhizosphere soil and leaves, though a weak positive correlation was found between rhizosphere soil and bark or litter. Seasonal analysis showed a strong positive correlation between yeast abundance in spring and summer, but no association between autumn and spring.

**Conclusion:**

Ethiopian forests are home for various yeast species including the stress-tolerant wild yeasts. This study highlights the significant yeast diversity in Ethiopian forests, with potential applications in improving industrial fermentation processes that operate under stressful conditions.

## 1. Introduction

Yeasts are a diverse group of microorganisms that occupy a vast array of ecological niches. These include plant substrates such as bark, leaves, flowers, and fruits, as well as non-plant environments like soil, air, water, and the surfaces of animals. ^1^ While significant numbers of research have focused on interactions between bacteria and plants, the study of yeast-plant relationships has received much less attention. Forest ecosystems, particularly, are rich in yeast diversity due to the presence of varied substrates like plant litters and forest soil. ^1, 2^ Wild yeasts inhabiting natural forest ecosystems display remarkable diversity in terms of morphology, color, and ecological roles. ^3– 5^ However, despite the invaluable importance of yeasts in ecological functions and biotechnology, understanding their distribution patterns and the factors governing their abundance remains incomplete, especially in complex ecosystems like forests.

The geographic locations and environmental conditions in different regions of the globe significantly influence the composition and structure of yeast communities. Forest ecosystems often host higher densities and numbers of yeast species compared to other habitats due to their diversity in plant and soil substrates. However, many factors such as climate, geography, biota, and natural disturbances affect their distribution and abundance. ^1^ Studies conducted in Jimma and Iluabaor zones of southwest Ethiopia suggest that the diversity of yeast communities is influenced by substrates such as soil, plant parts, and litter. Similarly, climate is a key global determinant of yeast abundance, with tree species, soil texture, and vegetation cover playing important roles. ^5^ Despite these insights, gaps persist in our understanding of yeast dynamics in forest ecosystems, particularly in natural forests of southwestern Ethiopia.

Yeasts are not only ecologically significant but are also important in industrial biotechnology. Wild yeasts have been used for millennia in food and beverage fermentation and are now essential in processes such as ethanol production, microbial oil synthesis, and single-cell protein production. ^6– 9^ Their roles as decomposers and research models further underscore their importance in both natural ecosystems and biotechnological applications. ^2^ However, characterizing wild yeasts for use in industrial fermentation remains challenging. The phenotypic characterization of yeasts involves testing their ability to metabolize diverse carbon sources, their tolerance to alcohol, and their survival under extreme environmental conditions like high or low pH and temperature. ^10– 12^ Previous studies, including our own preliminary findings, have demonstrated the potential application of wild yeasts of forest origin in ethanol production. ^13^

Overall, despite the ecological and biotechnological significance of wild yeasts, there is a notable lack of scientific reports on the distribution and physiological diversity of wild yeasts associated with different substrates in the natural forests of southwestern Ethiopia. This gap in knowledge hampers efforts to conserve yeast diversity and predict future changes in yeast populations within forest ecosystems. Addressing this knowledge gap is crucial for the scientific community, notably the mycologists, and industries relying on novel microbes isolated from natural resources. Therefore, the present study aims to assess the diversity, distribution, and phenotypic characterization of wild yeasts isolated from selected forests in southwest Ethiopia.

## 2. Methods

### 2.1 Study area and sample collection

A total of 200 samples of bark (n = 66), leaves (n = 38), rhizosphere soil (n = 54), and leaf litter (n = 42) were collected from natural stands of trees from three separate regions of Jimma ( *Belete-Gera* and *Boter Bacho*) and the Iluabaor ( *Yayo Biosphere Reserve*) zones. Briefly, bark and leaf samples were collected from surfaces of different plant species of the forest using sterile polythene bags. Bark samples of old logs of trees were collected from 0 to 3 m height of the logs at different gradients of the stem base, whereas the rhizosphere soils were collected at a depth of 0–10 cm from underneath the selected old logs after the collection of leaf litter samples. The study was conducted from September 2021 to June 2022.

The three regions ( *Belete-Gera *, *Yayo* Biosphere Reserve, and *Boter Bacho*) of Afromontane moist forests under study shared the following 23 different populations of tree species ( Table 1): *Albizia grandibracteata, Syzygium guineense, Ficus exasperata Vah, Milletia ferruginea, Croton macrostachyus, Sapium ellipticum, Schefflera abyssinica, Ritchiea albersii, Albizia gummifera, Ficus sycomorus, Ekebergia capensis, Acacia abyssinica, Diospyros abyssinica, Trichilia dregeana, Albizia malacophylla, Pinus Patula, Polyscios fulva, Acokanthera shimperi, Olinea rochetiniana, Ficus vasta, Catharanthus roseus, and Cordia africana.* The randomly chosen old trees in the plots were used to gather rhizosphere soil and plant substrates, such as bark, green leaves, and litter.

**Table 1.** Season of sample collection, plant spp. included at each sampling site, and number of yeast isolated from plant substrates.

Seasons	Location	Local name of Plant spp	*Scientific Name*	Number of yeasts and sources				total
				B	L	RS	Li	
Autumn	*Belete-Gera *	*Baddeessaa*	*Sysygium guineense*	4	3	0	0	7
		*Hororoo*	*Ekebergia capensis*	0	1	1	0	2
		*Hambabeessa*	*Albizia gummifera*	4	2	6	3	15
		*Bottoo*	*Schefflera abyssinica*	5	2	1	2	10
		*Harbuu*	*Ficus sycomorus*	2	3	0	3	8
		*Qaariyoo*	*Polyscios fulva*	1	4	1	4	10
		*Waddeessaa*	*Cordia Africana*	5	4	2	3	14
		*Qayee*	*Olinea rochetiniana*	6	0	1	0	7
Summer		*Deqoo*	*Ritchiea albersii*	10	2	11	6	29
		*Lookoo*	*Diospyros abyssinica*	3	4	6	8	21
		*Alalee*	*Albizia grandibracteata*	0	1	6	0	7
		*Waddeessa*	*Cordia Africana*	4	Ns	0	0	4
		*Cayii*	*Celtis Africana*	1	0	9	1	11
		*Balaantaa’ii*	*Ficus exasperata Vah*	10	0	12	4	26
		*Harbuu*	*Ficus sycomorus*	10	3	9	3	25
	*Yayo biosphere reserve*	*Hambabeessa*	*Albizia gummifera*	0	Ns	3	0	3
		*Luyyaa*	*Trichilia dregeana*	7	Ns	3	0	10
		*Qararoo*	*Acokanthera shimperi*	3	Ns	0	0	3
		*Qilxuu*	*Ficus vasta*	5	Ns	0	0	5
Spring	*Botor-Bacho *	*Laaftoo*	*Acacia abyssinica*	8	1	4	6	19
		*Waddeessa*	*Cordia Africana*	3	1	5	3	12
		*Paanaspachullaa*	*Pinus Patula*	6	0	4	3	13
		*Qilxuu*	*Ficus vasta*	4	5	1	4	14
		*Muka Jabo*	*Albizia malacophylla*	8	Ns	0	0	8
		*Bosoqa*	*Sapium ellipticum*	6	0	3	0	9
		*Alalee*	*Albizia grandibracteata*	3	3	0	0	6
		*Badeessa*	*Sysygium guineense*	4	0	2	0	6
		*Rukeessa*	*Catharanthus roseus*	5	7	3	0	15
		*Birbirsa*	*Milletia ferruginea*	8	1	0	0	9
		*Harbuu*	*Ficus sycomorus*	4	2	3	5	14
Autumn	*Yayo biosphere reserve*	*Luyyaa*	*Trichilia dregeana*	5	1	5	2	13
		*Balaantaa’ii*	*Ficus exasperata Vah*	8	4	2	0	14
		*Waddeessa*	*Cordia Africana*	0	0	4	1	5
		*Alalee*	*Albizia grandibracteata*	2	Ns	3	0	5
		*Birbirsa*	*Milletia ferruginea*	2	3	2	1	8
		*Qilxuu*	*Ficus vasta*	5	1	3	0	9
		*Baddeessa*	*Sysygium guineense*	1	1	2	0	4
		*Hambabeessa*	*Albizia gummifera*	1	0	3	2	6
		Total	*-*	163	59	120	64	406
		Std. Deviation	***-***	**25.054**	**7.764**	**19.314**	**11.094**	
		Mean	***-***	**7.77**	**2.37**	**5.95**	**3.43**	

Where, Ns = not sampled, B = Bark, L = Leaf, RS = Rhizosphere Soil, Li = Litter.

### 2.2 Yeast isolation and identification techniques

The methods used in this study for yeast isolation and identification were adapted from our previously established protocols. ^14^ Briefly, four grams of the solid samples (leaf, bark, and litters) were cut into smaller pieces before being added to the enrichment medium containing 1% yeast extract (Oxoid), 2% peptone (Oxoid), and 2% D-glucose (Fisher Chemical), along with 1 M HCl from 37% HCl (Fisher Chemical) in a 45 mL capacity flask. Each sample was cultured for 7 to 14 days at 30°C until signs of the beginning of fermentation were noticed. This step facilitated the growth and enrichment of indigenous yeast populations present in the samples. All the chemicals and media used in this study are products of Sigma-Aldrich (Oxoid Limited, USA) and HiMedia, India, unless specified otherwise.

**(a). Isolation of yeasts**

After the enrichment phase, actively fermenting cultures were diluted and plated onto yeast extract peptone dextrose (YPD) agar, supplemented with 0.2 g/L chloramphenicol (HiMedia, India) to inhibit bacterial growth. Approximately 100 μL of each diluted culture was spread on the surface of YPD agar plates. The plates were incubated at 30°C for 2 to 3 days, allowing yeast colonies to develop. Colonies exhibiting yeast-like morphology were selected for further analysis. A total of 406 wild yeast isolates were obtained from 200 environmental samples, including tree bark (163 isolates), rhizosphere soil (120 isolates), leaf litter (64 isolates), and leaves (59 isolates) collected from three distinct forest regions: Belete-Gera forest (54 isolates), Yayo Biosphere Reserve (80 isolates), and Boter-Bacho forest (66 isolates) ( Tables 1 and 2). The steps detailed below were followed rigorously to ensure optimal recovery and identification of yeast strains.

**Table 2.** Sampling period, sampling sites, and number of yeasts collected from tree bark, leaf, leaf litter and rhizosphere soil, south-western Ethiopia.

Seasons	Geographic coordinates of sampling sites	study sites	Sample types and No. of samples				Total samples	Sample types and No. of yeast isolates				Total yeast isolates	Isolates identified by MALDI TOF
			B	L	Li	Rh. S		B	L	Li	Rh. S		
Autumn	0855280E, 0197222N, 1989	BG	20	12	10	12	54	30	19	12	15	76	8
Summer & Autumn	808376 E, 926551 N, 1380	YBR	26	12	21	21	80	72	19	28	76	195	35
Spring	08.34844N,37.22900E, 1974	BB	20	14	11	21	66	61	21	24	29	135	17
Total			66	38	42	54	200	163	59	64	120	406	**60**

Where, BG-Belete Gera, YBR - Yayo Biosphere Reserve, BB- Botor-Becho, B –Bark, L-Leaf, Li- Litter, Rh. S – Rhizosphere Soil.

**(b). Colony purification and microscopic examination**

Isolated colonies were inoculated into 10 mL of sterile YPD broth for subsequent purification. After an additional growth phase, the cultures were streaked onto fresh YPD agar plates for purification. Morphology was observed after incubation, and colony purity was confirmed using phase-contrast microscopy at 100x magnification. Cultures showing characteristic yeast cell morphology (unicellular, budding cells) were deemed pure.

**(c). Preservation of yeast isolates**

For long-term storage, saturated cultures of pure yeast isolates were mixed with sterile 50% glycerol (Sigma Aldrich) and stored at -70°C for future analysis and characterization.

**(d). Genus-level identification**

To classify the yeast isolates to the genus level, a combination of biochemical, morphological, and physiological assays was employed. The Wikerham medium, which contained peptone (10 g/L), yeast extract (5 g/L), phenol red (HIMEDIA) (24 mg), and distilled water (1 L), was used to assess the yeast isolates’ capacity to ferment sugars (Fisher Chemical), such as glucose, galactose, fructose, maltose, lactose, xylose, and sucrose, using the standard procedure recommended by Zaid et al. ^15^ At a concentration of 2% (w/v), the sugars were dissolved. Each isolate’s active yeast cells (about 0.1 mL) were injected separately into test media that had been produced in Durham tubes. The test media was then incubated for three days at 30°C without any agitation. ^14^

Following procedures described in Tadesse et al. ^14^ research, the yeast isolates were spread out separately on YPD agar and incubated at 37, 40, 42, 44, and 45°C for 72 hours in order to assess the temperature tolerance of ethanol-tolerant yeasts. It was confirmed whether the yeast isolates were growing by looking directly at the colonies that formed on YPD agar plates. Parts of actively developing yeast cultures (1x107 cells/ml) were transferred into YPD broth that had been adjusted to different glucose concentrations (i.e., 40, 50, and 60%) in order to measure the osmotolerance of the yeast isolates. The growth was then assessed using optical density measurements taken at 600 nm using a UV-Vis spectrophotometer (Analytik Jena, Germany) after they had been cultured for 72 hours at 30 °C and 150 rpm.

**(e). Species-level identification by MALDI-TOF MS**

Matrix-Assisted Laser Desorption Ionization-Time-of-Flight Mass Spectrometry (MALDI-TOF MS), Zybio EXS3000 (Zybio Inc., China), was used to further identify a subset of yeast isolates that exhibited prominent physiological or phenotypic characteristics down to the species level. As mentioned in brief in our earlier study, ^14^ one colony was pipetted off the plate into a 1.5 ml tube (Eppendorf, Germany) and well mixed with 300 ml of water. Following a 2-minute centrifugation at 15,500 g with the addition of 900 ml of 100% ethanol, the mixture was discarded along with the supernatant. The pellet was left at room temperature to air dry for an hour. After that, the pellet was thoroughly combined with 50 milliliters of 70% v/v formic acid and then added to 50 milliliters of acetonitrile. For two minutes, the mixture was centrifuged again at 15,500 g. After applying one microliter of the supernatant to a section of the steel target, it was allowed to air dry at room temperature. As stated by Xiong et al., ^15^ one microliter of the matrix solution (cyano-4-hydroxycinnamic acid) was applied to each sample, and it was then left to air dry. A mass spectrometer, the EXS300 MALDI/TOF, was then used to make the measurements. The following categories were applied to the identifications using the manufacturer’s proposed modified score values: A score of 1.7 meant no identification, a score of >2 meant species identification, and a score of 1.7 to 1.9 meant genus identification.

### 2.3 Selecting the testing conditions

D-glucose (Fisher-Chemical, India), sodium chloride (NaCl) (NICE CHEMICALS LTD, India), and laboratory-grade 100% ethanol (SLC CHEMICALS DELHI, India) were used to choose the testing conditions. Using glucose concentrations ranging from 50 to 60% w/v, we assessed osmotolerance. Increasing concentrations of the stress factors were used to evaluate halotolerance (NaCl at 8, 10, and 12%) and ethanol tolerance (ethanol at 8, 10, 12, 14, and 16% v/v). Yeast Extract Peptone Dextrose (YPD) agar plates were utilized for the growth experiments. For the investigation of thermotolerance, isolates were grown on regular YPD agar without any test substance and then incubated at 37, 40, 42, and 45°C.

### 2.4 Data analysis

GraphPad Prism version 9.4.0 for Windows, developed by GraphPad Software, San Diego, California, USA ( www.graphpad.com), was used to create the heatmap plot and other graphs. Data were also analyzed using a repeated measures one-way ANOVA with a Geisser-Greenhouse’s epsilon correction to show a significant difference. Pearson’s correlation was used to evaluate correlations between sample substrates and seasons. The means were calculated, and significant differences between the means (p < 0.05) were evaluated using analysis of variance (ANOVA). A Venn diagram ( https://bioinfogp.cnb.csic.es/tools/venny/) was used to indicate the distribution of stress-tolerant yeast species in sample sources.

## 3. Results and Discussion

### 3.1 Yeast isolation and occurrence

Among the habitats explored for diversity and distribution of yeasts, plant surfaces, especially epiphytic yeasts on leaves and bark, showed the highest diversity. These findings are consistent with prior research, which reported the prevalence of yeasts in soil, marine environments, wildflowers, and even honey bee colonies. ^16– 19^ Among the collected samples, the highest yeast abundance was observed in bark samples (163 isolates), followed by soil (120 isolates) ( Table 2). This supports the assumption that yeasts colonize plant surfaces more prolifically than other habitats, possibly due to the diverse and dynamic microhabitats available in the phyllosphere. Bark, in particular, provides favorable niches for yeast proliferation, mainly due to its moisture retention and shelter from extreme environmental factors. ^20^

Geographical location might also play a significant role in shaping the yeast community structure. The *Yayo Biosphere Reserve* in the Iluabaor Zone yielded the most diverse range of wild yeast genera, followed by the *Boter-Bacho* Forest in Jimma Zone ( Table 2). This geographic variation in yeast isolation rates highlights the influence of local environmental conditions, such as temperature, humidity, and substrate composition, on yeast biodiversity. ^1^

Our results show that different substrates, including soil and phyllo-plane, harbor distinct yeast communities, suggesting substrate specificity in yeast colonization. This is in line with findings from previous studies, which reported that substrate type significantly influences yeast distribution patterns. ^16– 19^ For example, the yeast genera associated with soil were more diverse than those on the phyllo-plane, possibly due to the soil’s richness in organic matter and its ability to serve as a reservoir for microbial life. By examining the relationships between yeast genera and their respective habitats, we provide new insights into the ecological roles and dispersal strategies of these microorganisms.

### 3.2 Morphological characteristics of forest-derived wild yeasts

**Figure 1.**
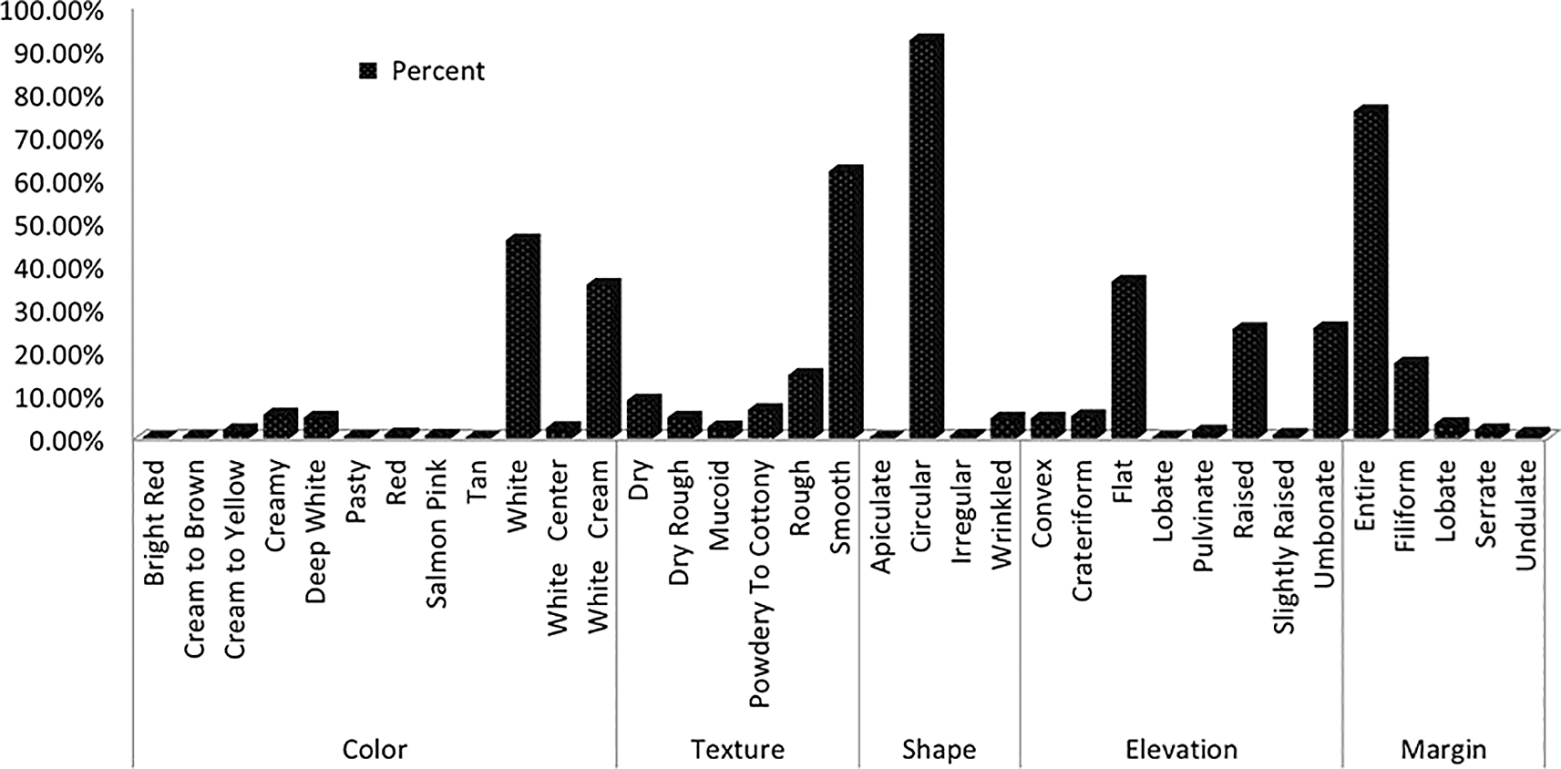
Colony morphology profile of wild yeasts isolated from natural forest.

Yeast colonies grown on YPD medium were classified into distinct phenotypes based on observable morphological traits. Predominant macroscopic characteristics included smooth (62%) or rough (14%) colony textures, circular (92.4%) or wrinkled (4.7%) shapes, smooth (75.9%) or filiform (17.5%) margins, and coloration ranging from white (46%) to cream (5.6%) or white-cream (35.7%) ( Figure 1).

**Figure 2.**
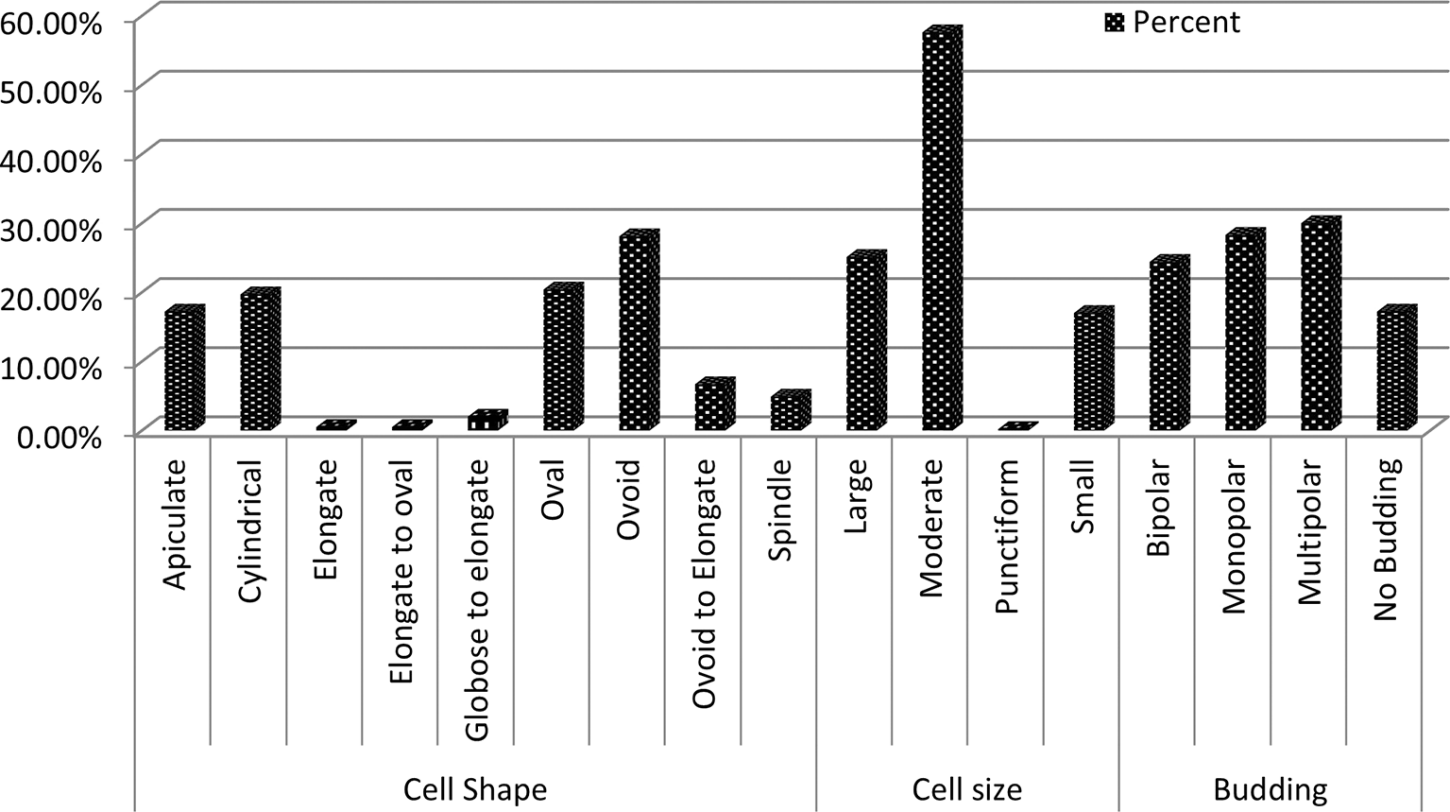
Microscopic morphology profile of wild yeasts isolated from natural forest.

Microscopic examination revealed diversity in cell shape and size, with apiculate (17.2%), round (28.1%), and oval (20.3%) shaped cells being most common. Cell sizes varied between small (17%), medium (57.6%), and large (25.1%), and the majority of cells reproduced by budding (82.7%), while the remaining cells (17.2%) did not exhibit budding ( Figure 2). Further analysis of wild yeast isolates from diverse sources displayed some characteristics, including creamy white pigmentation, smooth or rough colony texture, opacity, convex elevation, and entire or undulating margins. Other cells exhibited ellipsoidal, ogival, spherical, or oval morphologies, results consistent with findings from prior studies. ^21^

### 3.3 Physiological and biochemical analysis

**Figure 3.**
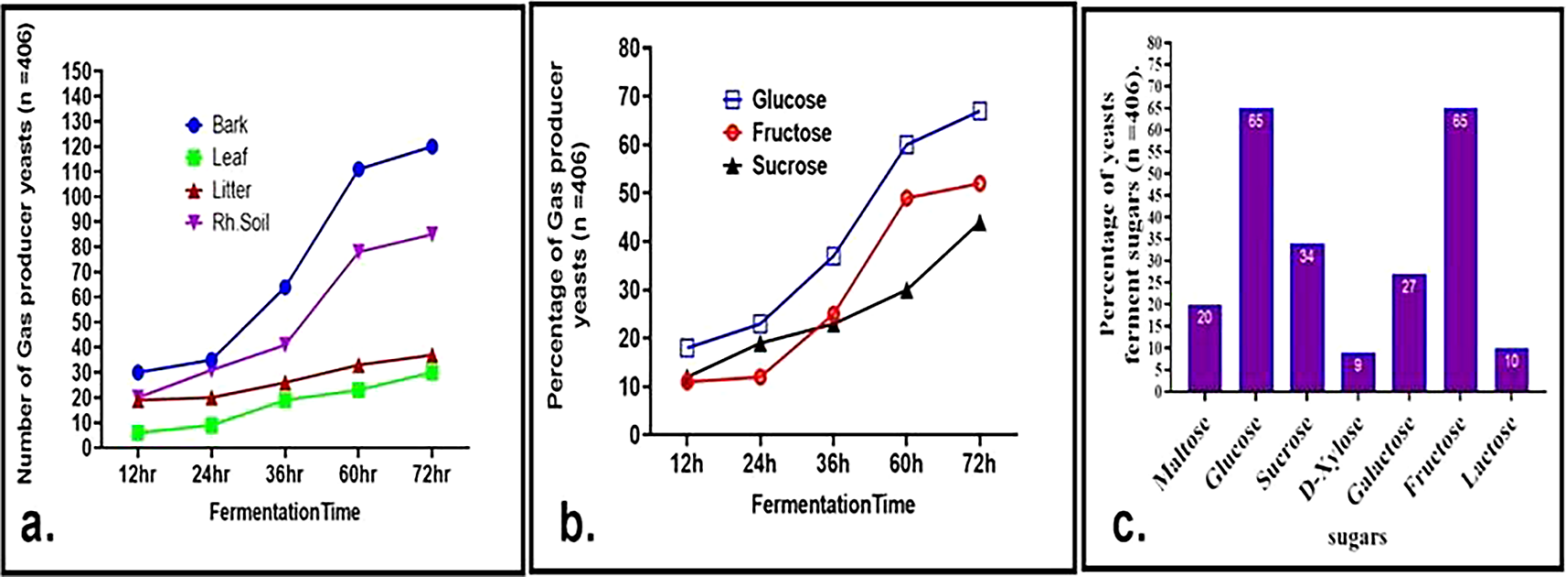
Fermentation profile of wild yeasts. a. Evaluation of gas-producing wild yeasts and the duration of fermentation in glucose cultures derived from soil, litter, and bark. b. Percentage of gas producing yeasts at different fermentation times from glucose, fructose, and sucrose. c. percentage of sugar fermenter wild yeast isolates cultured on different substrates after 72 h of incubation.

Physiological and biochemical profiling, including sugar fermentation, tolerance to ethanol salt, and growth temperature, was conducted on 406 yeast isolates, classified based on their morphological characteristics. Substrate fermentation was assessed over 72 hours, with measurements taken at 12-hour intervals. Among tested carbon sources, D-glucose supported the most rapid yeast growth and fermentation, with about 50% of the isolates fermenting D-glucose, a crucial trait in biotechnological applications. Hexose transporters (Hxt) and high-affinity glucose transporters (Hgt), with up to 20 distinct transporters contributing to rapid glucose metabolism in yeast cells, might facilitate glucose uptake. ^22^ Isolates screened from bark samples displayed the highest fermentation rates, followed by those from rhizosphere soil, while tree leaf isolates showed relatively slower growth and fermentation ( Figure 3a). Notably, 18% of the isolates initiated glucose fermentation within 12 hours. Additionally, 27% and 34% of the isolates were capable of fermenting galactose and sucrose, respectively ( Figure 3b and c), indicating possession of the gene responsible for the transport of sugars. GAL2 genes are known to play a critical role in galactose transport. ^23^ After 72 hours, 52% of the isolates fermented fructose, surpassing sucrose fermentation at 44%, which is consistent with previous findings by Camargo et al. and other researchers. ^22– 24^ About 23% of the isolates could ferment sucrose, aided by the enzyme invertase.

Approximately 20% of wild yeasts were able to ferment maltose, suggesting their potential applications in brewing and baking industries. Maltose ^25^ metabolism requires a proton gradient and expression of maltose permease for efficient carbohydrate utilization. ^25^ Additionally, 65% of the isolates fermented both glucose and fructose separately, while only a smaller proportion fermented lactose (10%) and xylose (9%) ( Figure 3c). The ability to ferment xylose, a crucial substrate for biofuel production, highlights the industrial relevance of these yeasts. These results significantly surpass previous findings by Camargo et al. ^24^ and other researchers ^26^ demonstrating broader metabolic versatility among wild yeast isolates, particularly in lactose, xylose, and maltose fermentation. Wild yeast strains that can ferment xylose are becoming increasingly crucial as lignocellulosic substrates are used to produce ecological fuels. ^27, 28^

### 3.4 Distribution of dominant yeast isolates in different samples

**Figure 4.**
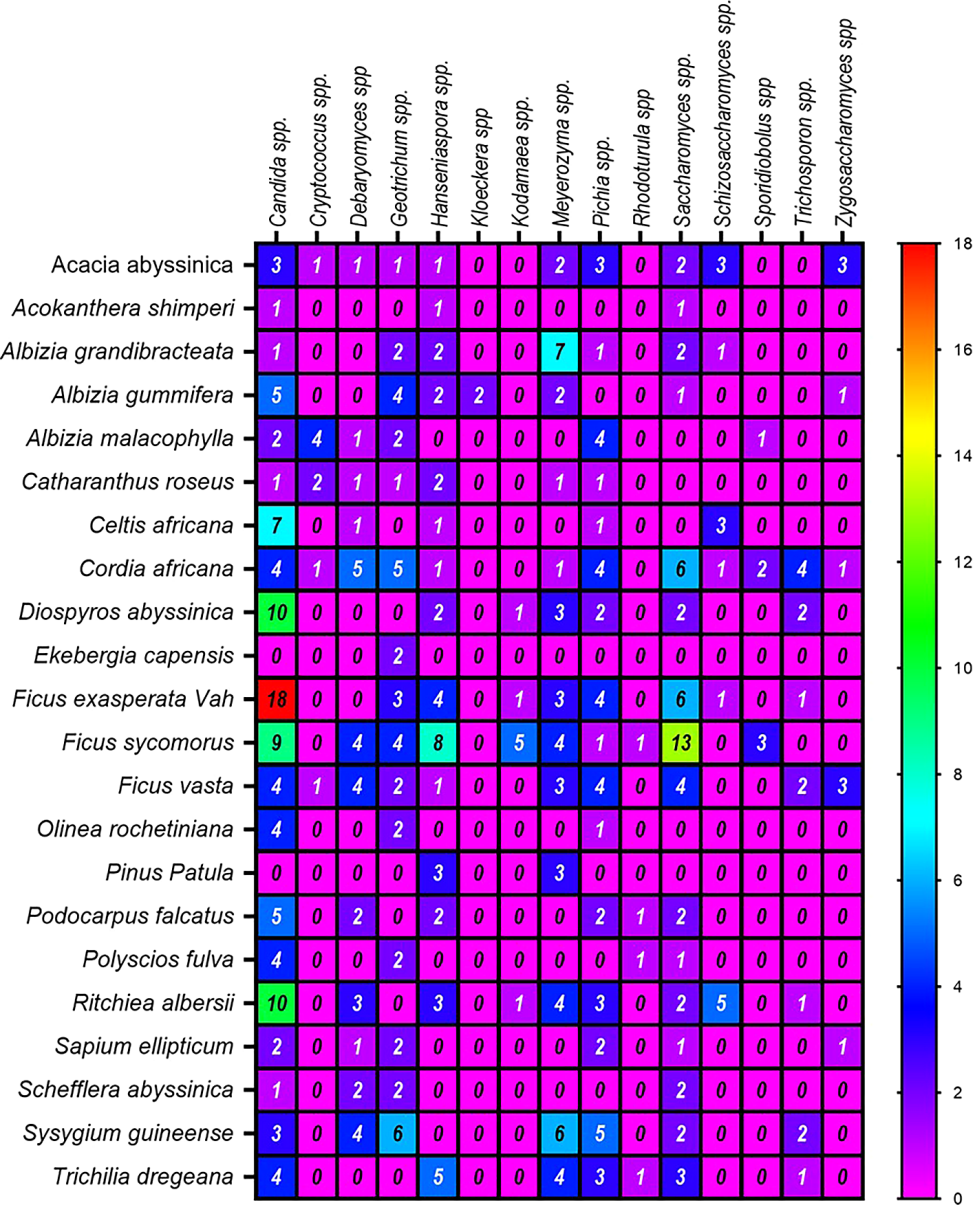
A Heatmap displaying the distribution and abundance of yeast in the examined substrates collected from various plant species.

In this study, a total of 406 yeast isolates were screened from various plant surfaces and rhizosphere soils and identified using morphological, physiological, and biochemical characteristics following the standard procedure suggested by Yarrow, ^29^ before categorizing them into 15 genera. Among these genera, four were basidiomycetes (Cryptococcus spp., *Rhodotorula* spp., *Sporidiobolus* spp., and *Trichosporon* spp.), while 11 were ascomycetes ( *Candida* spp., *Kodamaea* spp., *Meyerozyma* spp., *Pichia* spp., *Saccharomyces* spp., *Geotrichum* spp., *Kloeckera* spp., *Rhodotorula* spp., and *Hanseniaspora* spp.). Our research contradicted the conventional wisdom that ascomycetous yeasts were more common and abundant in agricultural soils, orchards, and grasslands. ^30^ Although ascomycota were the more diverse genera, their abundance was significantly higher than that of basidiomycetes ( Table 3 and Figure 4).

**Table 3.** Distribution and abundance of yeasts at different sampling points of tree barks, rhizosphere soil, litters and leaves samples.

Sample Location	Sample Size	Sample Sources	* Candida spp.*	* Cryptococcus spp.*	* Debaryomyces spp.*	* Geotrichum spp.*	* Hanseniaspora spp.*	* Kloeckera spp.*	* Kodamaea spp.*	* Meyerozyma spp.*	* Pichia spp.*	* Rhodoturula spp*	* Saccharomyces spp.*	* Schizosaccharomyces spp.*	* Sporidiobolus spp*	* Trichosporon spp.*	* Zygosaccharomyces spp*	Total
*Belete-Gera * Forest	20	Bark	8	1	3	6			1				5		1	4	1	30
	12	Rh. Soil	5		1	3		1		2			3					15
	10	Litter	5			4					2		1					12
	12	Leaf	6			8			1		1	1				2		19
Tot.1	54		24	1	4	21		1	2	2	3	1	9		1	6	**1**	**76**
*Botor-Becho * Forest	20	Bark	8	7	5		3			13	9		13				3	61
	21	Rh. Soil	7		4	6	4			4	3	1					1	30
	11	Litter			5	2	2			3	2		4	3			3	24
	14	Leaf	3		5	2					7	1	1		1	1		21
Tot. 2	66		18	7	19	10	9			20	21	2	18	3	1	1	**7**	**135**
*Yayo* Biosphere Reserve	26	Bark	15	1	1	1	17		2	9	4	1	12	3	3	3		72
	21	Rh. Soil	16		4	5	11		3	8	11		8	8		1	1	76
	21	Litter	17			1		1	1	2	1		3			1		27
	12	Leaf	6		2	2	1			3	1		2		1	1		19
Tot.3	80		54	1	7	9	29	1	6	22	17	1	25	11	4	6	**1**	**195**
Grand Total (Tot.1 + Tot.2 + Tot.3) = over all (%)	200		98 (24 %)	9 (2.2%)	29 (7.14 %)	40 (9.85 %)	38 (9.34 %)	2 (0.5 %)	9 (2.2 %)	44 (11 %)	41 (10.1%)	4 (1 %)	50 (12.32%)	14 (3.4 %)	6 (1.5 %)	13 (3.2 %)	9 (2.2 %)	**406 (100 %)**

Regarding the frequency of identification of yeast genera, the highest numbers were derived from the plants *Ficus vasta* (52 isolates), *Cordia africana* (41), *Ritchiea albersii* (35), *Ficus exasperata* Vah (32), and *Ficus sycomorus* (28) ( Figure 4). In contrast, the lowest genera of yeast were counted from *Ekebergia capensis* and *Acokanthera shimperi.* Among the dominant genera associated with the above plants was *Candida* spp. On the other hand, ascomycetes were the most abundant yeasts in *Ficus exasperata* Vah, while *Saccharomyces* spp. and *Meyerozyma* spp. were prevalent in *Ficus sycomorus* and *Albizia grandibracteata*, respectively. ^1, 31, 32^ The least common genera across all plant samples were *Kloeckera* spp. and *Rhodotorula* spp. (basidiomycetes), each representing only 0.5% of the total isolates ( Table 3, Figure 4) in agreement with previous reports. ^20, 33^ Yeast diversity also varied by source, with rhizosphere soil, bark, leaf, and litter samples showing different compositions. Across all sampling sources, the most frequently isolated genera were *Candida* spp. (24%), *Saccharomyces* spp. (12.3%), *Meyerozyma* spp. (10.8%), *Pichia* spp. (10%), *Geotrichum* spp. (9.9%), and *Hanseniaspora* spp. (9.4%) ( Table 3). These genera were isolated from almost all plant samples, though their relative abundances varied. The dominance of *Candida* spp. (98 isolates, 24%) in all plant sources except for litter highlights its widespread occurrence. Previous studies, such as Rao et al. ^34^ and Koricha et al., ^14^ have similarly isolated yeast taxa, including *Pichia, Candida*, and *Rhodotorula,* from fruits and tree barks.

*Saccharomyces* spp. were predominantly isolated from the bark samples of *Ficus sycomorus* (Harbuu), *Syzygium guineense* ( *Baddeessa*), *Ficus vasta* ( *Qilxuu*), and *Cordia Africana* ( *Wadeessa*), with the highest numbers from *Botor-Becho sites* (13 isolates) and *Yayo* Biosphere Reserve (12 isolates) ( Table 3). *Saccharomyces* spp. were absent from the leaf samples of *Belete-Gera* and the rhizosphere soil of Botor-Becho. This genus, commonly associated with locally fermented foods and beverages as practiced by humans, ^35^ is often found on oak trees, damaged fruits, and tree bark ^36^ and plays a key role in industrial applications such as ethanol production, enzyme synthesis, and feed fermentation. ^37^

*Meyerozyma* spp., notably *Meyerozyma guilliermondii*, were prevalent in the bark samples from *Botor-Becho* (13 isolates) but absent from the leaves and litter of *Belete-Gera* and *Botor-Becho* ( Table 3). *Meyerozyma* spp. is known for their efficiency in xylose fermentation, with a conversion rate of 85%, making them valuable for xylitol production from lignocellulosic materials. This yeast has been isolated from various environments such as tree bark, decaying wood, and soil.

*Pichia* spp. were identified across all sample sources except for bark and rhizosphere soil from Belete-Gera. It was more prevalent in the rhizosphere soil of Yayo Biosphere Reserve (11 isolates) and the bark of *Botor-Becho* (9 isolates) than in other sample sources. *Pichia* spp., particularly *Pichia kudriavzevii*, are notable for their potential in microbial oil production, single-cell proteins, ethanol production, and phytase activity. ^8, 38, 39^ Isolates, such as *P. kudriavzevii* KVMP10, are recognized for their thermotolerance, showing promise for their application in high-temperature fermentation processes. A number of thermotolerant yeast species, including *Pichia kudriavzevii* KVMP10 ^40^ and *P. kudriavzevii* KKU–TH33 and KKU–TH43, ^41^ were isolated from natural habitats and have been identified.

*Geotrichum* spp. and *Candida* spp. were the dominant genera isolated from *Belete-Gera *, while *Meyerozyma* and *Pichia* spp. were most prevalent in *Botor-Becho* ( Table 3). At *Yayo* Biosphere Reserve, *Candida* spp., *Hanseniaspora* spp., and *Saccharomyces* spp. were the most dominant isolated genera, with frequencies of 27.7%, 14.9%, and 12.82%, respectively. *Geotrichum* spp., commonly found on fruits and leaves, are linked to soil and can be spread by insects and other environmental factors. Among the commonly identified yeast species, *Candida* species, such as *C. shehatae*, are used in xylose fermentation. ^42– 44^

### 3.5 Stress-tolerant yeast species distribution among sample sources

**Figure 5.**
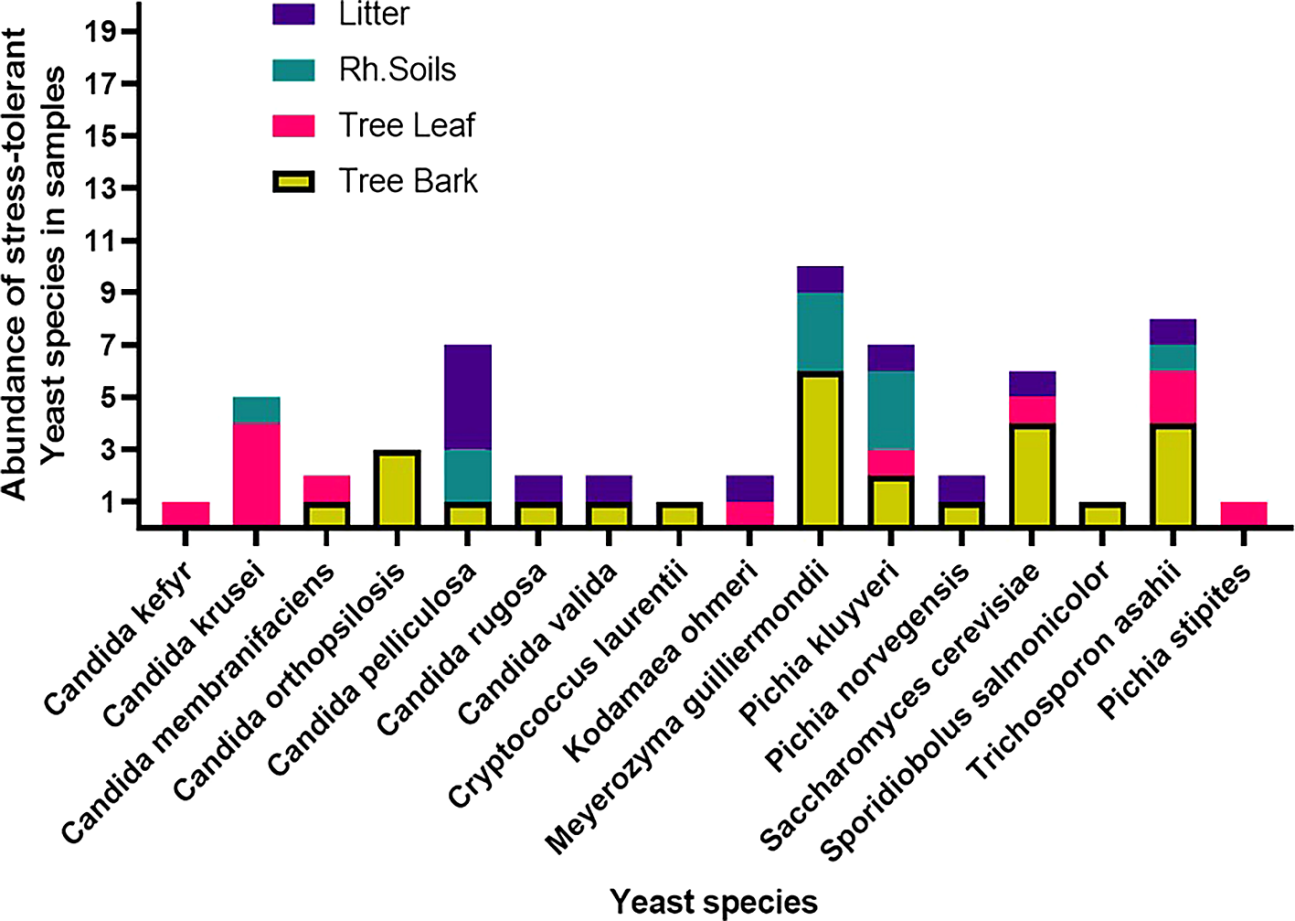
Distributions of stress-tolerant species across different sample sources.

Among the 60 stress-tolerant species identified using MALDI-TOF MS technology, the dominant ones are *Meyerozyma guilliermondii* (10 isolates), *Candida pelliculosa* (7), *Trichosporon asahii* (8), *Pichia kudriavzevii* (7), and *Saccharomyces cerevisiae* (6) ( Figure 5 **).** These species were widely distributed across various sample types. As reported earlier, the non-conventional yeasts, known for their stress tolerance, were represented by *Saccharomyces, Schizosaccharomyces, Dekkera, Pichia, Pachysolen, Kluyveromyces, Candida*, and *Meyerozyma.* ^21, 45^ The bark of *Sysygium guineense* ( *Baddeessa*), *Ficus vasta* ( *Qilxuu*), *Ficus sycomorus* ( *Harbuu*), and *Cordia Africana* ( *Wadeessa*) were key sources of *S. cerevisiae.*

**Figure 6.**
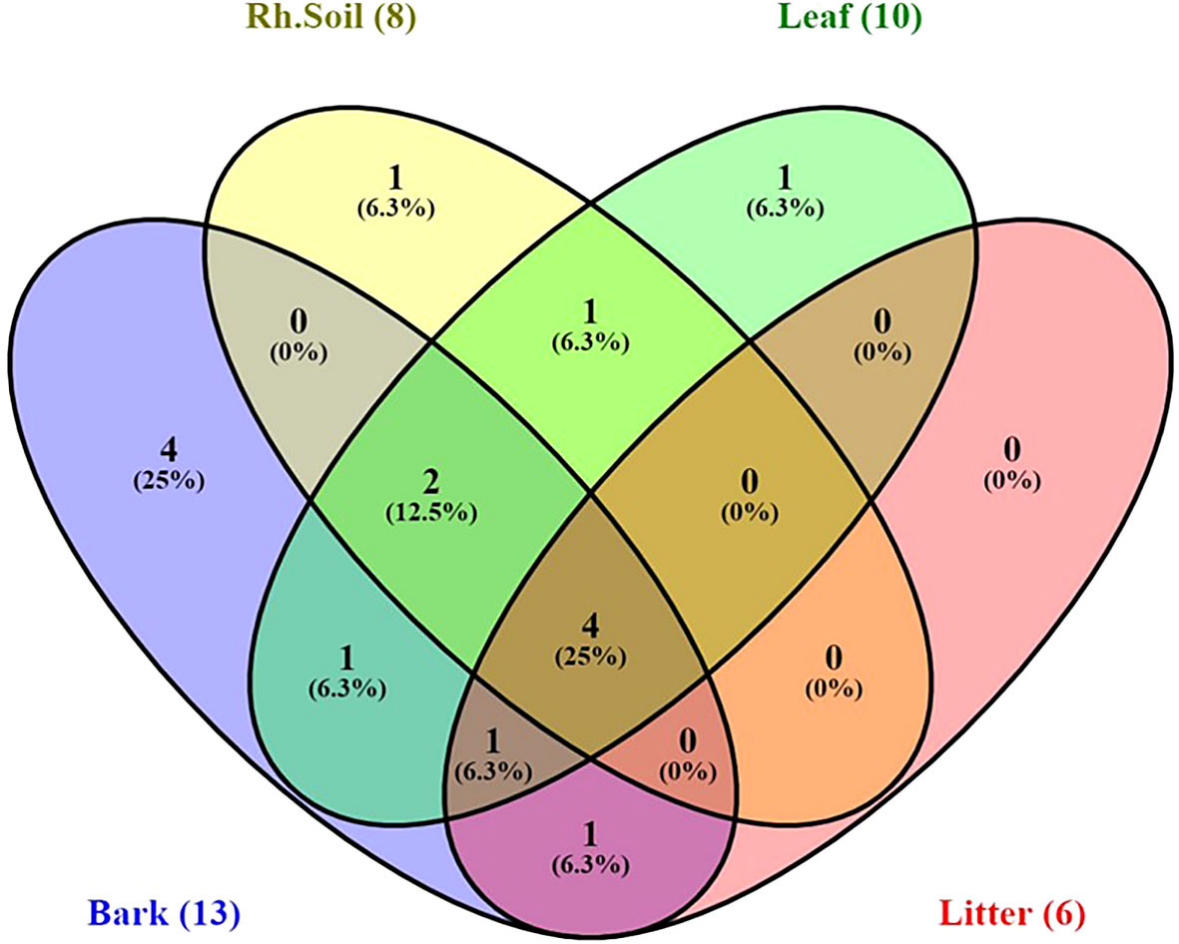
Venn diagram indicating distribution of stress-tolerant yeast species in sample sources, where, Rh.soil = Rhizosphere soil.

Yeast species distribution varied across substrates. For instance, 25% of the species were exclusively associated with bark, and 6.3% each with rhizosphere soil and leaves. Species commonly found across all substrates except leaves were *S. cerevisiae*, *M. guilliermondii, Pichia norvegensis*, *T. asahii,* and *C. pelliculosa.* Notably, *Pichia kluyveri* and *C. krusei.* *Kodamaea ohmeri* was specific to leaves and leaf litter, while bark, leaf, and litter shared *P. norvegensis* and *C. valida* ( Figure 6).

### 3.6 Seasonality of yeast distribution and abundance

**Figure 7.**
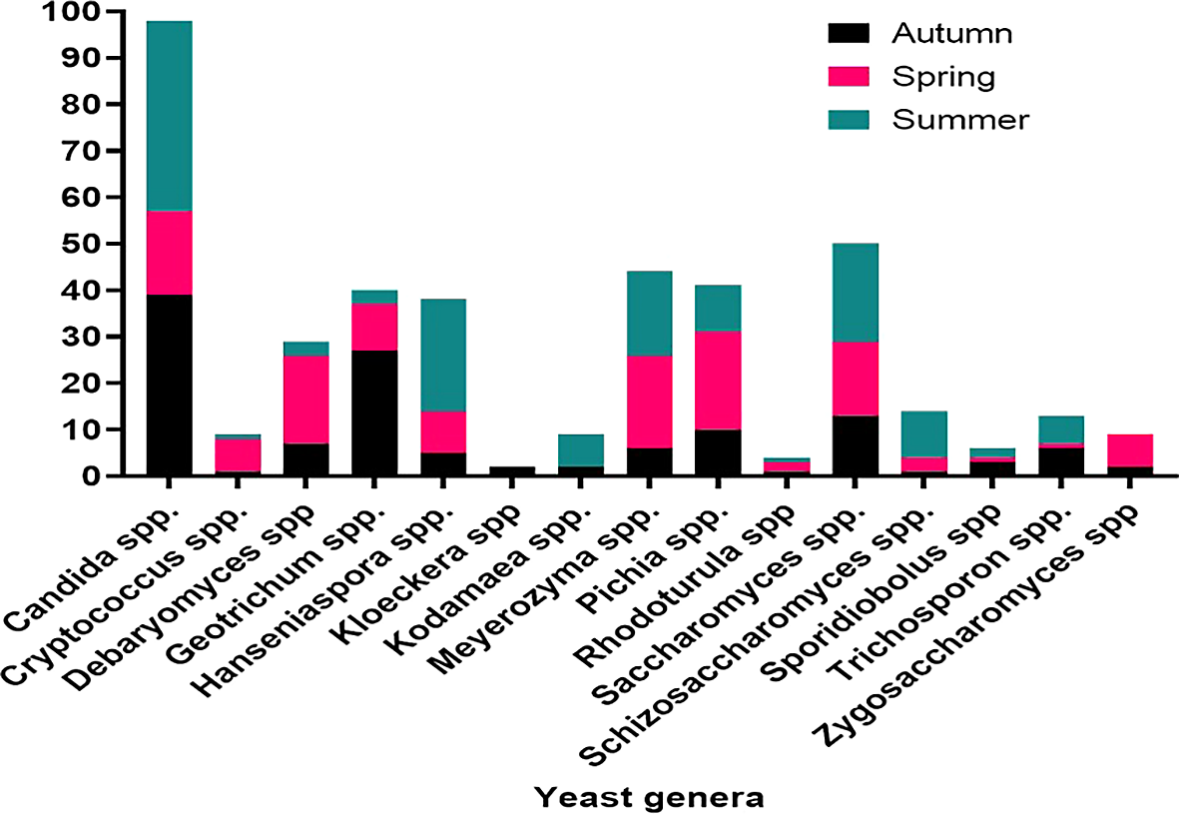
Seasonality in the distribution of dominant yeasts isolates.

The distribution of yeast isolates varied significantly across seasons, with the highest number of isolates recorded during the summer. Specifically during the summer season, 29 yeast isolates were obtained from *Ficus sycomorus*, 25 from *Ficus exasperata* Vah, and *Ritchiea albersii.* Additionally, 19 isolates were recorded from *Cordia africana* in autumn and 23 from *Ficus vasta* in the spring. Accordingly, summer yielded the most common yeast species, namely *Candida* spp. (41 isolates), followed by *Hanseniaspora* spp. (24) and *Saccharomyces* spp. (21). In autumn, *Candida* spp. (39) and *Geotrichum* spp. (27) dominated the isolated yeast population, while in spring, *Meyerozyma* and *Pichia* spp. were the most frequently encountered yeast species ( Figure 7).

Seasonal variations in yeast abundance were also significant. While no correlation was observed between autumn and spring, a strong correlation was found between autumn and summer (r = 0.79, P = 0.009). Spring and summer also showed a favorable correlation (r = 0.53, P = 0.043), indicating seasonal fluctuations in yeast distribution. Seasonal variations, plant species, soil type and depth, and site characteristics were identified as key factors influencing yeast diversity and abundance. ^44^ Deciduous and evergreen leaves, along with their respective environments, greatly impact seasonal shifts in the composition of leaf-associated yeast communities. ^33^ Some of these leaf yeasts enter the soil community as leaves decompose, although their numbers decrease significantly. Seasonal variations in yeast populations, such as a rise from 10 ^5^ to 10 ^7^ per gram of pasture grass leaves, have been observed, with the highest numbers occurring in summer and the lowest in winter, as noted by Robinson et al. ^46^ Temporal shifts in yeast communities on tree bark have been documented in temperate forests, though it remains unclear whether these patterns are seasonal or random. ^3, 47^

### 3.7 Relationships between study areas and yeast abundance as well as sample sources

**Figure 8.**
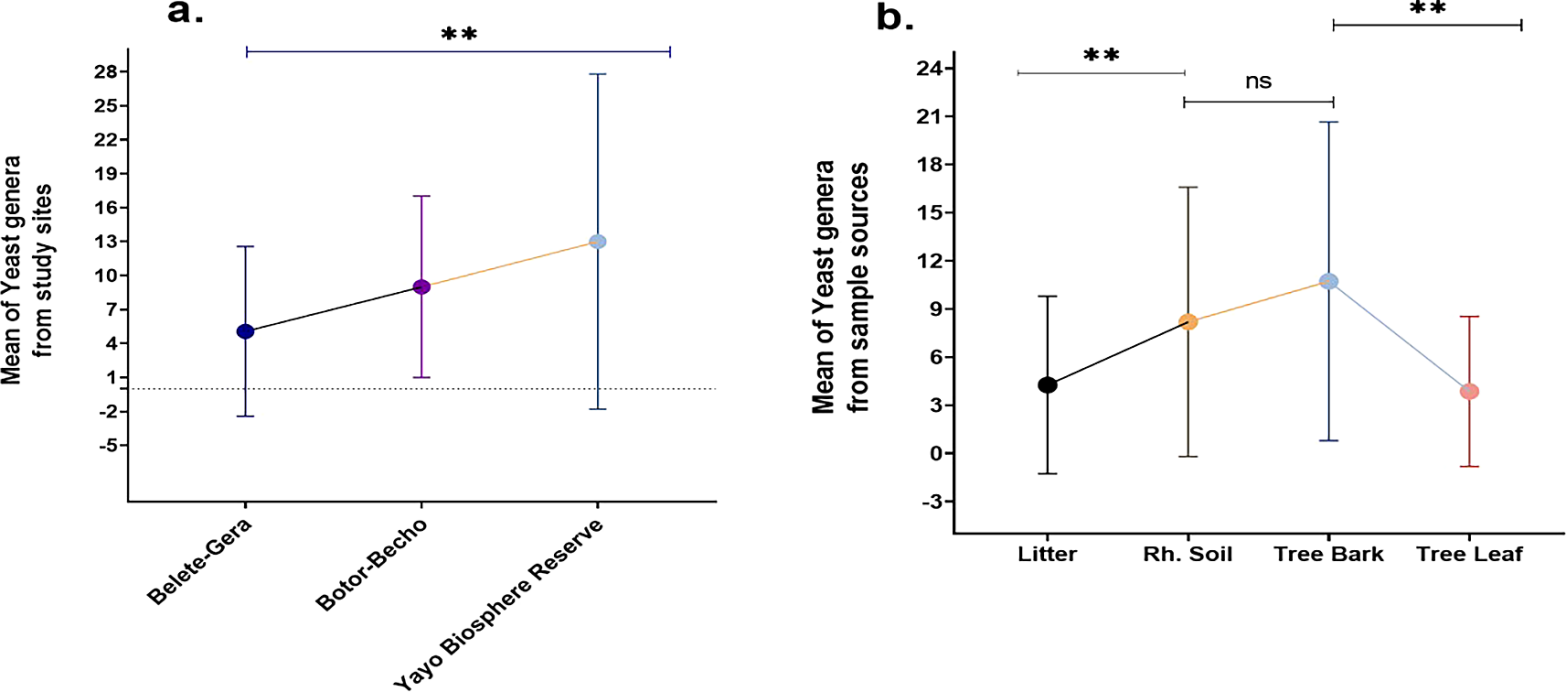
Mean of yeast genera from study sites (a) and sample sources (b).

This study explores the impact of various factors, including seasons, sample type, sample location, and host plant quality, on the abundance and diversity of wild yeast isolates. Tree bark was found to harbor the highest mean abundance of yeasts (10.7), while a weak positive correlation between yeast abundance in soil and bark (r = 0.326, P < 0.040) was observed. No correlation was found between yeast presence in bark and leaves (r = 0.064, P < 0.697), but significant differences between bark and leaf samples (t39 = 5.19, P < 0.00) were identified. The study also showed a significant positive correlation between yeast abundance in the rhizosphere soil and litter (r = 0.370, P = 0.019), confirming the interaction between soil and litter. The data indicated that rhizosphere soil and leaves differed significantly in yeast abundance (t _39_ = 3.293, P = 0.002), while no correlation was detected between bark and litter or leaves and litter ( Figure 8).

The *Yayo* Biosphere Reserve yielded the highest mean number of yeast genera (13), demonstrating a significant difference in yeast populations between study sites. Across the study locations, the mean abundance of yeast genera was significant ( Figure 8a). There was a noticeable difference in the mean yeast genus abundance between the bark and leaf and between the rhizosphere soil and litter. But there was no significant variation between the rhizosphere soil and the bark ( Figure 8b). The structure of yeast communities in litter was found to be strongly influenced by the predominant tree type, with the litter contributing secondary metabolites that act as nutritional sources for specific yeast species. These metabolites were proposed to be the primary factor influencing yeast composition, rather than the nutrient content. ^48^

The study also highlights that microbial communities vary between litters with identical nutritional content but differing origins, with soils beneath litter supporting a more diverse yeast community compared to those under logs. Tree bark in natural forests was identified as a rich habitat for stress-tolerant Ascomycetes species, resistant to adverse laboratory conditions, which may play a role in nitrogen cycling and community resilience in variable moisture conditions.

### 3.8 Analysis of the tolerance profile of wild yeasts

Table 4 displays the wild yeasts’ tolerance profile to various factors. Eighteen isolates from Boter-Becho sites in the spring and eleven from *Yayo* Biosphere Reserve sites in the summer were the highest isolates of wild yeast that could tolerate 42 °C from tree bark. Eight isolates of wild yeast from summertime litter at *Yayo* Biosphere Reserve locations had the highest tolerance rate, able to withstand 16% of ethanol. Fifty-one wild yeast isolates from tree bark collected in the spring at Boter-Becho sites had the maximum ability to tolerate glucose, at 60%. In spring, 11 isolates from Boter-Becho sites’ tree bark and leaf and 14 isolates from *Yayo* Biosphere Reserve sites’ tree bark throughout summer were the greatest number of wild yeast that could tolerate 12% salt concentration.

**Table 4.** Parameters for yeast tolerance, sample locations, sources, collection season, and total number of yeasts tolerated by growth-tested factors.

Parameters	Range	Sites															
		*Belete-G *				*Botor B*				*Yayo*							
		Sources				Sources				Sources							
		Litter	Rh. Soil	Tree Bark	Tree Leaf	Litter	Rh. Soil	Tree Bark	Tree Leaf	Litter		Rh. Soil		Tree Bark		Tree Leaf	
		Autumn	Autumn	Autumn	Autumn	Spring	Spring	Spring	Spring	Autumn	Summer	Autumn	Summer	Autumn	Summer	Autumn	Summer
Temperature	37°C	11	13	22	15	18	19	43	21	4	7	9	14	13	14	8	2
	40°C	3	3	5	6	6	8	18	10	4	5	4	9	2	11	5	1
	42°C	2	1	5	4	5	8	18	9	1	5	2	9	1	11	2	
Ethanol	8%	4	3	8	6	6	7	8	12	2	22	6	51	2	49	1	8
	10%	3	3	7	5	1	5	4	4	2	22	5	51	2	49	1	8
	12%	2	2	4	3		5	4	4	2	22	3	48	3	49	2	7
	14%	2	1	3	1			1	3	2	9	4	3		5	2	
	16%		1							2	8	2	3		1	2	
Osmotic pressure	30%	10	13	29	16	24	30	58	20	6	22	21	48	20	50	9	8
	40%	10	11	29	16	24	29	58	20	6	21	21	45	17	48	9	8
	50%	10	10	25	13	24	28	55	20	5	12	21	34	17	34	9	7
	60%	5	8	12	5	17	19	51	11	5	7	21	11	16	15	9	6
Nacl concentration	4%	12	14	28	19	23	31	53	21	6	20	21	54	17	47	10	8
	8%	5	2	12	8	22	26	49	19	5	9	15	31	7	29	7	3
	10%	4	2	6	5	5	4	12	11	3	4	9	21	3	24	5	1
	12%	4	1	4	4	5	3	11	11	3	4	4	10	1	14	3	1

**3.8.1 Ethanol tolerance of wild yeasts**

**Figure 9.**
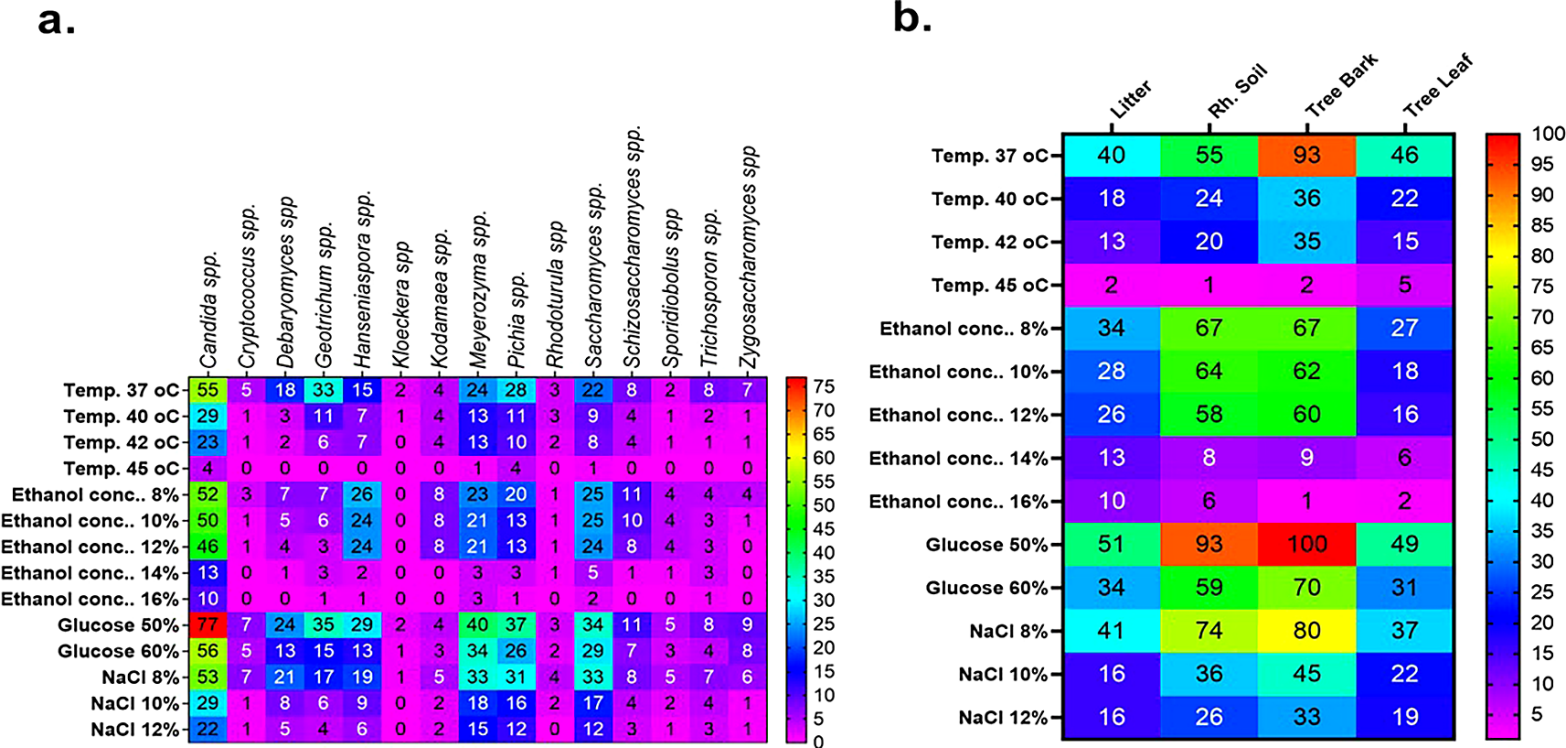
Heatmaps of the tolerance profile of 406 yeast genera isolated from natural forests (bark, rhizosphere soil, leaf, and litter).

Comparatively speaking, Candida spp. (n = 10) and *Meyerozyma* spp. (n = 3) exhibited greater resistance to high ethanol concentrations (16%) than other yeast genera. While the isolates from other genera only showed some inhibition at 12% ethanol, the majority of the latter genus isolates were severely inhibited at 14% and 16% ethanol. Particularly, the genera of *Candida* spp *, Meyerozyma* spp *, Saccharomyces* spp *,* and *Hanseniaspora* spp proved to be more resistant to this stress ( Figure 9a). Litter strains outperformed isolates from other origins in terms of average ethanol tolerance levels, whereas leaf strains showed significantly lower tolerance to all tested ethanol concentrations. The majority of ethanol-tolerant wild isolates at 16% ethanol concentration were found in litter, although isolates from rhizosphere soil and bark did not differ significantly in their ethanol tolerance of 8–12% of the ethanol concentration ( Figure 9b).

According to reports, several species in this genus have a high tolerance to ethanol and can even dominate industrial bioethanol fermentations and wine as contaminants. ^49^ This implies that achieving a greater ethanol titer through very-high-gravity fermentation could be an effective way to prevent these industrial contaminants. In yeast, ethanol decreases the growth, viability, and rate of fermentation of cells. A useful method for characterizing yeast species and strains that are being examined for use in alcoholic fermentations is to expose the yeast culture to ethanol at several concentrations until cellular growth inhibition occurs. This process allows one to determine the yeast’s tolerance to ethanol. Similar to other wild yeast isolates reported by, ^50^ we found that the growth of the isolates reduced with increasing ethanol concentration in the medium.

There were fewer tolerant genera of yeast in samples that were incubated at 30 °C when the ethanol concentration was raised from 8 to 16%. Figure 9a shows the results of a repeated measures one-way ANOVA with a Geisser-Greenhouse’s epsilon correction, which showed a significant change (P = 0.0260) of the number of tolerant yeasts to ethanol concentrations between genera of yeasts (columns) and a highly significant difference (P < 0.0001) between ethanol concentrations (rows). Additionally, the analysis revealed a significant difference (P = 0.0007) in the number of tolerant yeasts to ethanol concentrations between sample sources (columns) but no significant change (P = 0.0786) across rows, as illustrated in Figure 9b.

Remarkably, 50% w/v glucose was tolerated by at least some isolates of the majority of the assessed yeast genera. This is rather unexpected because many of those genera’ tested strains were isolated from environments without high sugar concentrations, which would seem to make them less likely to have developed tolerance mechanisms specific to osmotic stress. Consequently, these genera’s high general stress tolerance may be the cause of their tolerance to high sugar. It is commonly recognized that in many yeast species, the capacity to effectively transport glycerol into the cells is a crucial defense against osmotic stress. ^20, 51– 54^ It is therefore probable that the majority of these yeast taxa possess a strong route for both the manufacture and absorption of glycerol. However, because of the high osmotic pressure brought on by high glucose concentrations, a reduced rate of yeast cell multiplication was noted as the glucose rose. ^55^

Figure 9a shows the outcomes of a repeated measures one-way ANOVA with a Geisser-Greenhouse’s epsilon correction. The findings showed that there was no significant variation (P = 0.1233) in the number of yeasts that were tolerant to osmotic pressure across genera of wild yeasts (columns), but there was a significant difference (P = 0.0017) between rows (glucose concentration). Additionally, as Figure 7b illustrates, the study revealed no significant variation (P = 0.0959) in the number of tolerant yeasts to osmotic pressure between columns, but a significant change (P = 0.0149) across rows ( Figure 9b).

**3.8.2 Temperature tolerance of wild yeasts**

At 37, 40, 42, and 45 °C respectively, about 57, 24, 20, and 2.46 % of the isolates showed marked resistance to the assessed temperature. We have found several yeast genera that grow at 42 °C in this investigation. Specifically, when exposed to a high temperature, the majority of isolates of *Candida* (23), *Meyerozyma* (13), *Saccharomyces* (8), and *Pichia* (10) showed tolerance to the highest temperature tested (42 °C) ( Figure 9a). Based on sample sources, bark collected from natural forests was the source of isolation for the majority of the thermotolerant wild isolates ( Figure 9b). Interestingly, resistant isolates of Saccharomyces (1), Meyerozyma (1), Pichia (4), and Candida (4) were found to be tolerant at the maximum temperature (45 °C) that was tested. As shown in Figure 9b, out of all the 45 °C tolerant isolates mentioned above, five were isolated from tree leaves. The sudden changes in relative humidity and temperature that leaves experience could have an effect on the yeast population. One of the most prominent features of the leaf surface environment is that microorganisms have probably had to adapt to high fluxes of UV radiation. ^56, 57^ Because thermo-tolerant can reduce cooling costs, avoid bacterial contamination, and shorten the optimal temperature difference between enzymatic hydrolysis (45–50 °C) and fermentation (30–37 °C), a yeast that can ferment above 40 °C is chosen for usage in manufacturing environments. ^49^ Based on sample sources, bark collected from natural forests was the source of isolation for the majority of the thermotolerant wild isolates ( Figure 9b).

The results of a repeated measures one-way ANOVA with a Geisser-Greenhouse’s epsilon correction. The data indicated a highly significant difference (P<0.0001) between rows and no significant change (P = 0.0894) in the number of tolerant yeasts to temperature stress between genera of wild yeasts (across columns). Furthermore, as shown in Figure 7b, the analysis indicated a significant change (P = 0.0017) across rows but no significant variation (P = 0.1109) in the number of tolerant yeasts to temperature stress between sample sources (columns).

**3.8.3 Halotolerance (NaCl) of wild yeasts**

Figure 9a provides an overview of the salt tolerance for each yeast genus. About 94.5, 61, 29, and 24% of the isolates showed significant resistance at 4, 8, 10, and 12% of NaCl, respectively. To be more specific, most isolates *of Candida (*22), *Meyerozyma (15), Saccharomyces (12), Pichia (12),* and *Hanseniaspora spp. (6)* exhibited resistance when exposed to a high concentration of NaCl (12%) ( Figure 9a). For instance, strains of *Zygosaccharomyces* and *Schizosaccharomyces*, which were previously thought to be osmotolerant, were discovered to be susceptible to salt stress. This might point to a specific osmotolerance mechanism of these genera that fails to protect against ion toxicity brought on by salt stress at high sugar concentrations. It is vital to investigate how well yeasts tolerate high concentrations of salt (ionic stress) in commercial fermentations, as salt can promote yeast growth, improve ethanol production, and lower the danger of contamination by low-halotolerance microorganisms. ^50^ According to Mukherjee et al., ^58^ stress brought on by high concentrations of sodium chloride (NaCl) has been demonstrated to be more harmful from high intracellular cation concentrations than from hyperosmotic stress. Our findings supported previous observations ^54^ that yeast cell development is enhanced by a combination of mild temperature and salt stressors. On average, isolates with varying sample sources showed differing levels of tolerance towards NaCl. Accordingly, the majority of halotolerant wild isolates were found in rhizosphere soil and bark from natural forests; yeast genera from these sources had a higher tolerance to NaCl than litter and leaves.

The data indicated a highly significant difference (P < 0.0001) between rows and no significant change (P = 0.0894) in the number of tolerant yeasts to salt stress between yeast genera across columns ( Figure 9a). Additionally, Figure 9b displayed the distribution of NaCl stress-tolerant yeasts from various sources. The result revealed that while there was no significant variation (P = 0.0626) in the number of tolerant yeasts to NaCl stress between sample sources, there was a highly significant shift (P < 0.0001) across salt concentrations.

## Conclusions

This study provides a comprehensive analysis of the diversity, distribution and phenotypic characterization of yeast species in the rhizosphere soil and plant surfaces within a natural forest in southwestern Ethiopia. The findings revealed the presence of diverse yeast genera, including *Candida*, *Saccharomyces*, *Meyerozyma*, *Pichia*, *Geotrichum*, and *Hanseniaspora*, with the highest yeast isolation rates occurring from the bark of tree species such as *Qilxuu*, *Waddeessa*, *Deqoo*, *Baalantaa’ii*, and *Harbuu.* The prevalence of yeast isolates in tree bark suggests that it serves as a primary substrate for yeast colonization, while some genera were found to inhabit multiple plant hosts, indicating non-host-specific behavior.

The weak positive correlations between yeast abundance in rhizosphere soil and both bark and litter suggest some interaction between these substrates. However, there was no significant correlation between yeast counts in leaves and either bark or rhizosphere soil, highlighting the distinct microbial communities associated with different plant parts. Additionally, seasonal variations were observed, with spring and summer showing a strong positive correlation in yeast abundance, whereas autumn displayed no significant associations with other seasons.

The study identified several stress-tolerant yeast species, such as *Meyerozyma guilliermondii*, *Trichosporon asahii*, *Candida pelliculosa*, *Pichia kluyveri*, and *Saccharomyces cerevisiae*, which were capable of surviving under extreme conditions, including ethanol, heat, osmotic stress, and saline environments. These species were found across a variety of substrates, demonstrating their adaptability to different ecological niches.

In general, substrate type and geographic location significantly influence the distribution of yeast species in natural ecosystems. The identification of stress-tolerant yeasts with biotechnological potential further highlights the importance of natural forests as reservoirs for microbial diversity. These findings not only contribute to the understanding of yeast ecology but also offer a foundation for future bioprospecting efforts and ecological monitoring in similar environments.

## Credit authorship contribution statement

KB: Conceptualization, Formal analysis, Supervision, Manuscript review; TT: Conceptualization, Investigation, Formal analysis, Writing - original draft, Visualization; AD: Formal analysis, oversight, conceptualization, original draft review, visualization, and funding acquisition; DD: Research, Formal examination and composing a first draft.

## Data Availability

**Figshare**: Diversity, Diversity and Tolerance profile of Wild Yeasts from Natural Forests, https://doi.org/10.6084/m9.figshare.28078370. ^59^ The underlying data for this project includes the following:
• Distribution and abundance of wild yeasts in the examined substrates collected from the study areas and plant species. • Distribution of wild yeast isolates at different seasons. • Tolerance profile of wild yeast genera to particular stress conditions. Data are available under the terms of the Creative Commons Attribution 4.0 International license (CC-BY 4.0).
