# Supplementary material for: Diversity, Distribution, and Phenotypic Characterization of Cultivable Wild Yeasts Isolated from Natural Forest
Source: F1000Res. 2025 Apr 8;14:105. Originally published 2025 Jan 17. [Version 3] doi: 10.12688/f1000research.160250.3 (PMC12033980; doi:10.12688/f1000research.160250.3)
Supplement: Peer review history

# Reviewer report: Reviewer response for version 3

**DOI:** 10.5256/f1000research.179817.r376733

**Published:** 2025-04-26

**Authors:** Pablo Villarreal

**Affiliations:** 1 Facultad de Química y Biología, Departamento de Biología, University of Santiago Chile, Santiago, Santiago Metropolitan Region, Chile

I confirm that all the issues I raised in my previous review have been adequately addressed, and I have no further comments to make.

Is the work clearly and accurately presented and does it cite the current literature?

Yes

If applicable, is the statistical analysis and its interpretation appropriate?

Partly

Are all the source data underlying the results available to ensure full reproducibility?

Yes

Is the study design appropriate and is the work technically sound?

No

Are the conclusions drawn adequately supported by the results?

Partly

Are sufficient details of methods and analysis provided to allow replication by others?

No

Reviewer Expertise:

Microbial ecology

I confirm that I have read this submission and believe that I have an appropriate level of expertise to confirm that it is of an acceptable scientific standard.

# Reviewer report: Reviewer response for version 2

**DOI:** 10.5256/f1000research.178781.r370601

**Published:** 2025-03-21

**Authors:** Pablo Villarreal

**Affiliations:** 1 Facultad de Química y Biología, Departamento de Biología, University of Santiago Chile, Santiago, Santiago Metropolitan Region, Chile

The authors have addressed almost all of my concerns; however, the figures still require further improvement. There is no statistical analysis supporting the results, which is a major limitation. Additionally, Figure 8 is overly complex and does not effectively convey the data. I strongly recommend using a heatmap instead, as it would provide a more precise and more interpretable representation of the results.

Is the work clearly and accurately presented and does it cite the current literature?

Yes

If applicable, is the statistical analysis and its interpretation appropriate?

Partly

Are all the source data underlying the results available to ensure full reproducibility?

Yes

Is the study design appropriate and is the work technically sound?

No

Are the conclusions drawn adequately supported by the results?

Partly

Are sufficient details of methods and analysis provided to allow replication by others?

No

Reviewer Expertise:

Microbial ecology

I confirm that I have read this submission and believe that I have an appropriate level of expertise to confirm that it is of an acceptable scientific standard, however I have significant reservations, as outlined above.

# Response

**Published:** 2025-03-24

**Authors:** Teshome Tadesse

**Affiliations:** Biology, Mettu University, Metu, Oromia, Ethiopia

The comments are scientific and helpful. Based on these, we incorporated a description of the statistical analysis and converted Figure 8 into a heatmap in the text.

# Reviewer report: Reviewer response for version 1

**DOI:** 10.5256/f1000research.176114.r362747

**Published:** 2025-02-14

**Authors:** Lydia R Heasley

**Affiliations:** 1 Colorado State University, Fort Collins, Colorado, USA

In this article, the authors report on their studies of collecting and characterizing wild yeasts from diverse natural environments in South West Ethiopia. They identify multiple yeast species and associate them with environmental sources such as tree bark, leaf litter, etc. In addition, they define the metabolic and stress-tolerance profiles of many of their isolates. Overall, I find that the authors provide a clear presentation of their study, which contributes to important ongoing efforts to sample fungal biodiversity across the globe. I have no major critiques, but below I outline my minor critiques:

 1) If space allows, I think that including representative images/photographs of the different colony morphologies, colours, etc. would be valuable to better illustrate the data in Fig. 2.

 2) It would be great if the isolates collected could be sequenced using whole genome sequencing and analysed in order to identify whether these Ethiopian isolates represent unique clades of their respective species or if they share ancestry with other known clades within the population architecture. If WGS is not a technique available to the researchers, I understand the lack of these analyses. Either for this publication or in the future, I recommend finding a collaborator who can perform these analyses with the authors. As WGS data is now critical for integrating population diversity with species architecture, this would certainly increase the impact of the authors findings and the ability of other fungal evolution scientists to utilize and build on the authors work.

Is the work clearly and accurately presented and does it cite the current literature?

Yes

If applicable, is the statistical analysis and its interpretation appropriate?

Yes

Are all the source data underlying the results available to ensure full reproducibility?

Yes

Is the study design appropriate and is the work technically sound?

Yes

Are the conclusions drawn adequately supported by the results?

Yes

Are sufficient details of methods and analysis provided to allow replication by others?

Yes

Reviewer Expertise:

Yeast genomics and genetics; evolutionary genetics; genome stability.

I confirm that I have read this submission and believe that I have an appropriate level of expertise to confirm that it is of an acceptable scientific standard.

# Response

**Published:** 2025-02-27

**Authors:** Teshome Tadesse

**Affiliations:** Biology, Mettu University, Metu, Oromia, Ethiopia

We thank the anonymous reviewer for the very constructive and positive comments.

 Reviewer’s Suggestion: The importance of adding some pictures to enhance the readability of the manuscript:

 Author Response:

- Some pictures of the yeast isolates (morphology) as seen under the microscope and on solid media are included (Figures, 2 and 4). The same figures were incorporated into the manuscript.   

# Reviewer report: Reviewer response for version 1

**DOI:** 10.5256/f1000research.176114.r360818

**Published:** 2025-01-31

**Authors:** Pablo Villarreal

**Affiliations:** 1 Facultad de Química y Biología, Departamento de Biología, University of Santiago Chile, Santiago, Santiago Metropolitan Region, Chile

The manuscript by Tadesse et al., *Diversity, Distribution, and Phenotypic Characterization of Cultivable Wild Yeasts Isolated from Natural Forest*, presents an interesting and relevant study on the diversity and distribution of cultivable yeasts in rhizosphere samples and other plant-associated habitats from natural forests in Africa.

 This study could significantly contribute to the understanding of yeast ecology in a geographically underexplored region, highlighting the importance of these microorganisms in natural ecosystems. The data obtained through morphological, physiological, and biochemical analyses, combined with MALDI-TOF identification, are valuable and can be a reference for future studies. However, the manuscript has some limitations and areas that should be improved before indexing.

 One of the major concerns is the methodology used for yeast isolation, which introduces a significant bias throughout the study. Using an enrichment medium (pre-culturing the samples in YPD) selectively favors certain yeast species, which may skew the results and lead to overgeneralized ecological conclusions. For example, observing that more yeasts were isolated from bark than from soil could be a direct consequence of the isolation technique rather than an actual ecological pattern. Without a more unbiased approach, the findings should be interpreted with caution.

 Additionally, MALDI-TOF is not an entirely reliable method for species-level identification and should ideally be complemented with molecular marker sequencing, such as ITS or D1/D2, to confirm species assignments. The manuscript does not clearly explain the rationale behind sample selection and does not justify why more samples were collected from specific locations than others.

 This inconsistency extends to seasonal comparisons, where varying samples across different seasons could introduce biases. If higher yeast abundance is observed in a specific location or season, it may result from sampling inconsistencies rather than a proper ecological pattern.

 Furthermore, there is a lack of methodological details on how yeast growth was measured. It is unclear how growth curves were performed, what specific parameters were measured, and which kinetic factors were compared. These details are crucial for ensuring reproducibility and validating the conclusions drawn from the data. The figures presented in the manuscript also do not support the conclusions. A comparison of ODmax between isolates or growth rates could provide more robust evidence regarding stress tolerance among the yeast strains.

 In summary, while the manuscript presents valuable findings, it lacks methodological rigor in several key aspects. A major revision is necessary to clarify the methodology, improve data interpretation, and ensure the results fully support the conclusions. Addressing these issues will significantly enhance the manuscript’s impact and reliability.

Is the work clearly and accurately presented and does it cite the current literature?

Yes

If applicable, is the statistical analysis and its interpretation appropriate?

Partly

Are all the source data underlying the results available to ensure full reproducibility?

Yes

Is the study design appropriate and is the work technically sound?

No

Are the conclusions drawn adequately supported by the results?

Partly

Are sufficient details of methods and analysis provided to allow replication by others?

No

Reviewer Expertise:

Microbial ecology

I confirm that I have read this submission and believe that I have an appropriate level of expertise to state that I do not consider it to be of an acceptable scientific standard, for reasons outlined above.

# Response

**Published:** 2025-02-27

**Authors:** Teshome Tadesse

**Affiliations:** Biology, Mettu University, Metu, Oromia, Ethiopia

We are grateful to the anonymous reviewer for their insightful and encouraging remarks.

**Response 1. Using an enrichment medium (pre-culturing the samples in YPD). **Indeed, certain yeast species are preferentially favored when using an enrichment medium. The research successfully isolated stress-tolerant wild yeast for possible industry application. The study's goal was to understand yeast diversity in Ethiopian natural forests with an emphasis on enriching wild yeast populations with specific characteristics for biotechnological applications. It also isolated fermentative yeasts from environmental samples with stress tolerances, underscoring the significance of understanding yeast diversity for biotechnological applications.

**Response** **2**

  (MALDI-TOF is not an entirely reliable method for **species-level identification** and should ideally be complemented with molecular marker sequencing, such as ITS or D1/D2, to confirm species assignments)

 The comment is constructive and scientific. If the isolated Ethiopian yeasts were sequenced using molecular markers and analyzed to determine whether they belong to distinct clades of their species or share ancestry with other known clades within the population architecture, it would have been fantastic and made the study more comprehensive. This would undoubtedly boost the findings' impact and enable other researchers studying yeast evolution to use and expand on the current work. However, we didn’t have access to the technology could not go that far. We, however, believe that the current identification level will not compromise the validity of the findings.  

**Response 3**

  (The rationale behind sample selection and why more samples were collected from specific locations than others.)

**Rationale:** To isolate wild yeasts from undisturbed parts of southwest Ethiopia, which is primarily densely populated forest territory, plant substrates and the underlying soil were taken from natural forests. From the perspective of biotechnological applications, we focused on the bark of old trees to isolate yeasts with a variety of phenotypic traits linked to the bark of old trees and their surroundings in the natural forests.

**Why more samples were collected from specific locations than** others

 The availability of old logs in the Afromontane rainforests of southwest Ethiopia and the area coverage of forests in the chosen study areas are the main reasons why more samples were taken from some locations than others. Since the two zones are evergreen regions with nearly identical climatic conditions and a similar pattern of forest cover throughout Ethiopia, they were purposively chosen to enhance the chance of getting diverse yeast with potentially diverse industrial applications.

**Response 4**

**Measurement of yeast growth**

 (lack of methodological details on how yeast growth was measured. It is unclear how growth curves were performed, what specific parameters were measured, and which kinetic factors were compared)

 As mentioned in our previously published work ^13^, optical density measurements at 600 nm obtained with a spectrophotometer (Analytik Jena, Germany) both before and following incubation at 30°C for 48 hours were used to measure the growth (maximum optical density) of wild yeast under various conditions (tolerance to salt, ethanol, glucose, temperature and acidic). The same methods are presented in the manuscript.

**Response 5**

 The figures presented in the manuscript also do not support the conclusions. A comparison of ODmax between isolates or growth rates could provide more robust evidence regarding stress tolerance among the yeast strains

- The comment is constructive and scientific. After the comment, we added Figure 8 in the manuscript which demonstrated more robust evidence regarding the tolerance of stress-tolerant wild yeasts at different conditions after 48 hours in YPD broth that Optical density was measured at 600nm.
